# Increased Prevalence of Insulin Resistance and Metabolic Syndrome in Men With Early‐Onset Androgenetic Alopecia: A Case–Control Study

**DOI:** 10.1111/jocd.70458

**Published:** 2025-11-28

**Authors:** Orkun Erden, Ahu Yorulmaz, Basak Yalcin

**Affiliations:** ^1^ Department of Dermatology Atlas University Faculty of Medicine Istanbul Turkiye; ^2^ Department of Dermatology Ankara Bilkent City Hospital Ankara Turkiye; ^3^ Department of Dermatology Private Clinic Ankara Turkiye

**Keywords:** early‐onset androgenetic alopecia, insulin resistance, metabolic syndrome, young adult

## Abstract

**Aim:**

This study aimed to investigate the relationship between early‐onset androgenetic alopecia (AGA) and the prevalence of insulin resistance (IR) and metabolic syndrome (MetS) in young adult males.

**Methods:**

A total of 200 males aged 18 to 35 years were enrolled in this case–control study, including 100 patients with early‐onset AGA and 100 age‐ and body mass index‐matched controls. Clinical staging was performed using the Hamilton–Norwood classification, and all participants underwent standardized physical and biochemical evaluation.

**Results:**

The prevalence of insulin resistance (HOMA‐IR ≥ 2.7) was significantly higher in the AGA group compared to the control group (18% vs. 4%, *p* = 0.003). Similarly, MetS was more common among AGA patients (21% vs. 9%, *p* = 0.011). Fasting insulin levels, triglyceride concentrations, and HOMA‐IR scores were all significantly elevated in the AGA group, while HDL‐C levels were comparable between groups. Waist circumference was also significantly greater in the AGA group (*p* = 0.026). When stratified by AGA severity, the frequency of IR progressively increased with increasing Hamilton–Norwood stages. No significant differences were observed in fasting glucose or blood pressure categories. Although individual MetS components were similar, cumulative clustering of risk factors appeared to be associated with a significantly higher MetS frequency among patients with AGA.

**Conclusion:**

Early‐onset AGA in men may serve as a visible clinical indicator of underlying metabolic disturbances, particularly IR. Routine metabolic screening may be advisable in young men presenting with advanced AGA stage.

## 1 Introduction

Androgenetic alopecia (AGA) is the most common form of hair loss in men, characterized by progressive and permanent miniaturization of hair follicles, occurring on a genetic basis with increased androgen sensitivity. Clinically, it typically presents with thinning and hair loss in the frontal and vertex regions [1]. AGA is a widespread condition affecting approximately half of all men, and its prevalence is correlated with age [2]. The third decade of life is a common onset in most cases of AGA; however, some individuals experience early onset. This early form is referred to in the literature as “early‐onset” or “premature” AGA, commonly defined by Hamilton–Norwood (HN) stage III or higher hair loss occurring before the age of 35. Early‐onset AGA is more aggressive and can lead to balding at a younger age [3].

Recent studies have indicated that AGA may not merely be a cosmetic concern, but rather a clinical condition associated with various systemic and metabolic disorders. Particularly in early‐onset cases, significant associations have been reported between AGA and insulin resistance (IR), dyslipidemia, hypertension, metabolic syndrome (MetS), and cardiovascular diseases [3, 4]. Elevated levels of dihydrotestosterone and disruptions in insulin signaling are thought to be shared mechanisms involved in both AGA pathogenesis and metabolic dysfunction [5, 6]. Moreover, hyperinsulinemia secondary to IR may stimulate androgen synthesis and impair perifollicular microcirculation, contributing to follicular atrophy [7]. These findings suggest that AGA, especially when it presents at a young age, might have potential as a clinical marker of insulin resistance and cardiometabolic risk [8, 9].

The aim of this study was to investigate whether early‐onset AGA is associated with IR and MetS in young adult males, thereby assessing the hypothesis that AGA may serve as a clinical indicator of underlying systemic metabolic disturbances.

## 2 Methods

### 2.1 Study Setting and Ethics Approval

This single‐center case–control study was conducted at the Department of Dermatology, our hospital, between November 2014 and October 2015. The study protocol was approved by the local review board, and all procedures adhered to the principles outlined in the Declaration of Helsinki. All participants provided written informed consent prior to enrollment.

### 2.2 Study Population and Inclusion Criteria

A total of 200 male participants were enrolled, including 100 patients aged 18 to 35 years who presented with clinically diagnosed early‐onset AGA, and 100 male controls matched for age and body mass index (BMI). Controls were selected from the same outpatient clinic population and underwent dermatological scalp examinations. AGA diagnosis and staging were based on the HN classification, which ranges from stage I to VII. All assessments were performed by a single dermatologist to minimize inter‐observer variability.

Participants were included if they were male, aged between 18 and 35 years, and had undergone examination for AGA. Patients in the AGA group had to have HN stage III or higher, while controls had stage I or II. Exclusion criteria included: history or diagnosis of diabetes mellitus, impaired glucose tolerance, hypothalamic–pituitary–adrenal axis disorders, thyroid dysfunction, significant recent weight change, or use of medications that could affect glucose metabolism, lipid profiles, thyroid function, or hair physiology in the prior 6 months.

### 2.3 Clinical and Anthropometric Measurements

A detailed personal and family medical history was collected, followed by complete physical and dermatological examinations. Body weight and height were measured, and BMI was calculated. Waist circumference was measured at the midpoint between the lowest rib and the iliac crest while the participant was standing. Blood pressure was measured using a calibrated sphygmomanometer from the right arm after a minimum of 10 min of rest in a seated position.

### 2.4 Laboratory Analysis

Following a 12‐h overnight fast, venous blood samples were obtained. Fasting plasma glucose was measured by enzymatic colorimetric assay using the Hexokinase/glucose‐6‐phosphate dehydrogenase method on a Beckman Coulter AU5800 analyzer. Serum insulin levels were determined via chemiluminescent immunoassay using a sandwich technique with the manufacturer's ultrasensitive insulin kit on a Beckman Coulter DXI 800 analyzer. Triglycerides and high‐density lipoprotein (HDL) cholesterol levels were measured by enzymatic colorimetric assays employing lipoprotein lipase/glycerol kinase and cholesterol esterase/cholesterol oxidase reactions, respectively. All analyses were performed at the hospital's Biochemistry Laboratory using validated protocols.

### 2.5 Definitions and Diagnostic Criteria

IR was calculated using the Homeostatic Model Assessment for Insulin Resistance (HOMA‐IR) formula: HOMA‐IR = [Fasting Insulin (μIU/mL) × Fasting Glucose (mg/dL)] / 405. A HOMA‐IR value ≥ 2.7 was considered indicative of IR, based on validated literature cutoffs.

MetS was defined according to the 2006 International Diabetes Federation (IDF) criteria for European men [10]. Central obesity (waist circumference ≥ 94 cm or BMI ≥ 30 kg/m^2^) was required for diagnosis, along with fulfillment of at least two of the following: Triglycerides ≥ 150 mg/dL; HDL cholesterol < 40 mg/dL; Systolic blood pressure ≥ 130 mmHg or diastolic ≥ 85 mmHg; Fasting plasma glucose ≥ 100 mg/dL.

### 2.6 Statistical Analysis

The IBM SPSS software for the Windows operating system (Version 20.0) was employed for data entry, creation of preliminary tables, and analysis (SPSS Inc., Chicago, IL, USA). Continuous variables were expressed as mean ± standard deviation or median with minimum to maximum values, as appropriate. Categorical variables were summarized as frequencies and percentages. The Kolmogorov–Smirnov test was used to assess the normality of distribution in continuous variables. Student's *t*‐test or Mann–Whitney *U* test was used to compare continuous variables depending on distribution. Chi‐square or Fisher's exact tests were applied for categorical comparisons.

## 3 Results

The mean age of participants was 27.4 ± 4.2 years in the AGA group and 27.0 ± 4.5 years in the control group (*p* = 0.638). The comparison of anthropometric, clinical, and biochemical parameters between the AGA and control groups is presented in Table 1. Waist circumference was significantly higher in the AGA group compared to controls (92.43 ± 8.21 cm vs. 89.89 ± 7.64 cm, *p* = 0.026). Patients with AGA were significantly more likely to report a positive family history of the disease, particularly involving both maternal and paternal inheritance (31% vs. 5%; *p* < 0.001).

**TABLE 1:** Comparison of anthropometric, clinical, and biochemical parameters between androgenetic alopecia patients and healthy controls.

Variable	AGA (*n* = 100)	Control (*n* = 100)	*p*
Height (cm)	175.95 ± 6.39	175.95 ± 5.95	1.00
Body weight (kg)	78.01 ± 11.32	75.96 ± 11.14	0.207
BMI (kg/m^2^)	25.14 ± 3.01	24.48 ± 2.93	0.118
Waist Circumference (cm)	92.43 ± 8.21	89.89 ± 7.64	0.026
Systolic Blood Pressure (mmHg)	124.75 ± 10.39	122.44 ± 9.46	0.140
Diastolic Blood Pressure (mmHg)	74.48 ± 8.31	74.00 ± 8.24	0.508
Family history of AGA			
No	16 (16.0%)	59 (59.0%)	< 0.001
Paternal	36 (36.0%)	25 (25.0%)	
Maternal	17 (17.0%)	11 (11.0%)	
Maternal + Paternal	31 (31.0%)	5 (5.0%)	
FBG (mg/dL)	86.48 ± 10.95	86.53 ± 8.30	0.743
Insulin (μIU/mL)	11.66 ± 14.66	6.33 ± 3.36	0.005
TG (mg/dL)	139.97 ± 93.79	133.94 ± 139.41	0.043
HDL (mg/dL)	44.5 ± 9.6	45.5 ± 13.9	0.687
HOMA‐IR	2.52 ± 3.65	1.25 ± 0.76	0.013
Insulin Resistance			
Present	18 (18.0%)	4 (4.0%)	0.003
Absent	82 (82.0%)	96 (96.0%)	
Hamilton–Norwood classification			
I	—	79 (39.5%)	
II	—	21 (10.5%)	
III	55 (27.5%)	—	
IV	37 (18.5%)	—	
V	7 (3.5%)	—	
VI	1 (0.5%)	—	

Abbreviations: AGA, androgenetic alopecia; BMI, body mass index; FBG, fasting blood glucose; HOMA‐IR, homeostasis model assessment of insulin resistance.

Fasting serum insulin levels (11.66 ± 14.66 μIU/mL vs. 6.33 ± 3.36 μIU/mL, *p* = 0.005), triglyceride levels (139.97 ± 93.79 mg/dL vs. 133.94 ± 139.41 mg/dL, *p* = 0.043), and HOMA‐IR scores (2.52 ± 3.65 vs. 1.25 ± 0.76, *p* = 0.013) were significantly elevated in the AGA group. Consequently, the prevalence of IR (defined as HOMA‐IR ≥ 2.7) was significantly higher among patients with AGA (18%) than among healthy controls (4%) (*p* = 0.003).

The prevalence of MetS was higher in the AGA group compared to the control group (21% vs. 9%, *p* = 0.011). Notably, individual MetS components, despite showing marginal differences in values, were similar across groups. These included waist circumference ≥ 94 cm (36% vs. 30%, *p* = 0.452), systolic blood pressure ≥ 130 mmHg (37% vs. 24%, *p* = 0.065), diastolic blood pressure ≥ 85 mmHg (10% vs. 7%, *p* = 0.612), fasting blood glucose ≥ 100 mg/dL (9% vs. 6%, *p* = 0.591), HDL < 40 mg/dL (36% vs. 30%, *p* = 0.452), and triglycerides ≥ 150 mg/dL (30% vs. 24%, *p* = 0.426) (Table 2).

**TABLE 2:** Comparison of metabolic syndrome prevalence and diagnostic criteria between androgenetic alopecia patients and healthy controls.

Variable	AGA (*n* = 100)	Control (*n* = 100)	*p*
Metabolic Syndrome			
Present	21 (21%)	9 (9%)	0.011
Absent	79 (79%)	91 (91%)	
Waist Circumference ≥ 94 cm	36 (36%)	30 (30%)	0.452
Systolic BP ≥ 130 mmHg	37 (37%)	24 (24%)	0.065
Diastolic BP ≥ 85 mmHg	10 (10%)	7 (7%)	0.612
FBG ≥ 100 mg/dL	9 (9%)	6 (6%)	0.591
HDL < 40 mg/dL	36 (36%)	30 (30%)	0.452
TG ≥ 150 mg/dL	30 (30%)	24 (24%)	0.426

Abbreviations: AGA, androgenetic alopecia; BP, blood pressure; FBG, fasting blood glucose; HDL, high‐density lipoprotein; TG, triglycerides.

**FIGURE 1:**
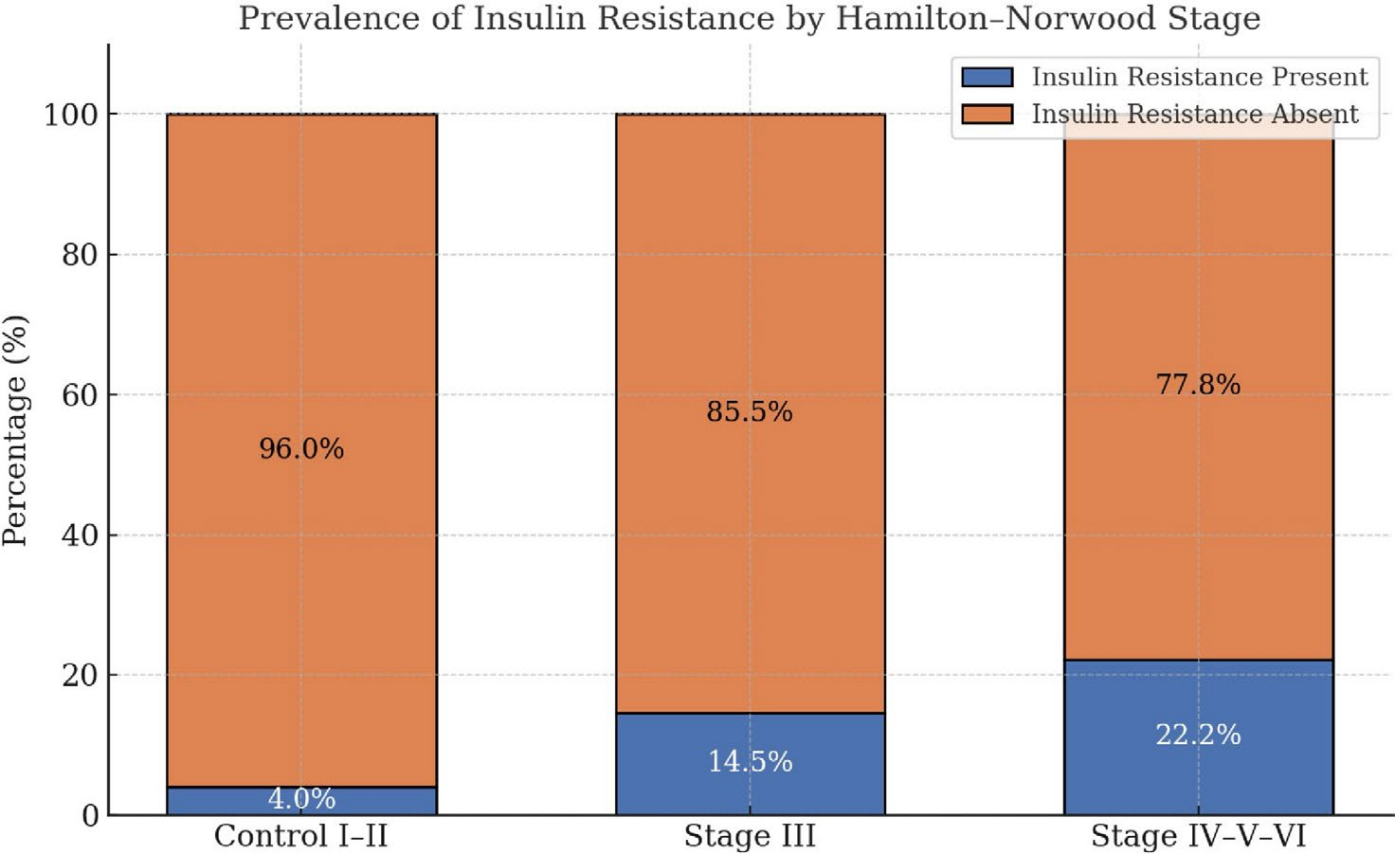
Prevalence of insulin resistance by Hamilton–Norwood stage.

The prevalence of IR was 4% in the control group (HN stage I–II), while IR was detected in 14.5% of the patients with stage III AGA, and in 22.2% of those with stage IV–VI AGA. This progressive increase was significant (*p* = 0.003) (Figure 1).

## 4 Discussion

The present examination of IR and MetS in young male patients with early‐onset AGA reveals strong associations between AGA and the frequency of IR and MetS in young males. Moreover, the frequency of IR increased progressively with greater AGA severity. These results suggest that AGA could potentially serve as an early clinical marker of underlying metabolic disturbances in young men.

The elevated prevalence of MetS in early‐onset AGA indicates that AGA could be an early manifestation of systemic metabolic abnormalities. Similar results have been reported in the literature, with several studies highlighting an increased risk of MetS among individuals with AGA and emphasizing the need for careful monitoring of abdominal obesity, dyslipidemia, and glucose metabolism disorders in these patients [11, 12, 13]. A recent comprehensive systematic review and meta‐analysis reported a 3.46‐fold higher prevalence of MetS in individuals with AGA (95% CI: 2.38–5.05) [5], while another large‐scale study documented this risk ratio as 2.81 (95% CI: 2.16–3.66) [14]. The early onset of AGA may therefore represent a potential clinical marker for the early identification of cardiometabolic risk [3]. Furthermore, theoretical models have proposed that the hormonal background of AGA and the pathophysiology of MetS may intersect via shared mechanisms such as androgen sensitivity, impaired insulin signaling, and chronic low‐grade inflammation [5, 15, 16, 17, 18]. Although the present study was not designed to directly investigate these mechanisms, our findings highlight the importance of systematic screening for MetS in individuals with early‐onset AGA as a potential opportunity for the early prevention of cardiovascular diseases.

In this study, IR (defined as HOMA‐IR ≥ 2.7) prevalence was greater in the AGA group, and an increasing trend in the frequency of IR was observed with advancing HN stages, suggesting a direct relationship between AGA severity and the presence of underlying metabolic dysfunction. The literature indeed shows that individuals with AGA, particularly those with early‐onset disease, tend to have a higher frequency of hyperinsulinemia and disturbances in glucose metabolism [4, 19]. Dysregulation in androgen metabolism, particularly elevated local levels of dihydrotestosterone, may contribute not only to the miniaturization of hair follicles but also to impairments in insulin signaling pathways [20, 21]. Additionally, perifollicular hypoxia resulting from endothelial dysfunction and vasoconstrictive effects may lead to microcirculatory impairment and subsequent follicular atrophy [22, 23]. Low‐grade chronic inflammation, adipokine imbalances, and oxidative stress associated with increased visceral adiposity are also thought to play critical roles in the pathogenesis of IR. These mechanisms may collectively contribute to hair loss as a consequence of systemic metabolic dysregulation in AGA [7, 17]. Sorour et al. reported notable associations between AGA and metabolic disturbance, with a particular emphasis on IR. They concluded that Galectin‐3 may serve as a potential biomarker or therapeutic target in the context of the AGA and IR relationship [24]. Furthermore, some studies have proposed a possible genetic link between these two conditions, further supporting a shared biological basis [25]. In line with these previous findings, our results support the hypothesis that AGA may reflect not only a dermatological issue but also a clinical manifestation of systemic IR.

Abdominal obesity is closely associated with IR as well as MetS and may be more prevalent among individuals with AGA, which is supported by the higher waist circumference values of our AGA group [26]. Visceral fat tissue is widely recognized as a major driver of various metabolic abnormalities, including IR, dyslipidemia, and chronic inflammatory responses. One study even identified waist circumference as the most important risk factor for MetS development in patients with AGA [15]. Numerous studies in the literature have also supported a positive association between AGA and abdominal obesity, reporting significant correlations between AGA severity and both waist circumference and body fat percentage [15, 27, 28]. However, it must be noted that studies reporting similar waist circumference values in AGA and healthy controls exist [29]. It has also been hypothesized that increased androgen receptor density in abdominal fat depots may facilitate regional fat accumulation, further strengthening the link between AGA and abdominal obesity [30]. In this context, waist circumference measurements in individuals with AGA may not only serve as a simple anthropometric parameter but may also be a clinical parameter to assess the overall metabolic risk profile.

Ours is a single‐center and cross‐sectional study, which restricts the interpretation of causal relationships between AGA and metabolic alterations. It also goes without saying that the results are only relevant for males. The study population consisted entirely of a young age group (18–35 years), and therefore, the relationships may not apply to older individuals, especially with respect to the differing impacts of comorbidities. The HOMA‐IR index is a widely accepted measure of IR; however, the gold standard method is the hyperinsulinemic‐euglycemic clamp technique, and further studies employing this approach to assess IR may provide more accurate data and outputs. Potential confounding factors such as dietary habits, physical activity level, and smoking status were not assessed. Nevertheless, matching the control group with patients based on age and BMI allowed for more accurate comparisons of baseline metabolic parameters.

## 5 Conclusion

The prevalence of IR and MetS is significantly higher in young adult men with early‐onset AGA compared to controls. Moreover, IR frequency increased in parallel with advanced AGA stage according to the HN classification. These findings suggest that early‐onset AGA may serve as a visible clinical marker of underlying metabolic disturbances. Given its easily recognizable presentation and early age of onset, AGA may offer a valuable opportunity for the early screening of insulin resistance and cardiometabolic risk factors, which may facilitate the implementation of preventive interventions.

## Author Contributions

O.E. conceptualized the study and designed it. O.E. contributed in the study design and data acquisition. O.E., A.Y., B.Y. contributed in data acquisition, data analysis and drafting the manuscript. O.E. and A.Y. contributed in data acquisition and manuscript drafting and editing. All authors have read and approved the final manuscript.

## Ethics Statement

The study protocol was approved by the Institutional Review Board of Ankara Numune Training and Research Hospital (Approval No: 2015‐939, Date: 28 January 2015), and all procedures adhered to the principles outlined in the Declaration of Helsinki. All participants provided written informed consent prior to enrollment.

## Conflicts of Interest

The authors declare no conflicts of interest.

## Data Availability

The data that support the findings of this study are available from the corresponding author upon reasonable request.
